# Effect of Biscuit Flour and Fermented Defatted “Alperujo” Co-Administration on Intestinal Mucosa Morphology and Productive Performance in Laying Hens

**DOI:** 10.3390/ani11041075

**Published:** 2021-04-09

**Authors:** Néstor Porras, Agustín Rebollada-Merino, Carmen Bárcena, Francisco J. Mayoral-Alegre, Juan Manuel Lomillos, Lucas Domínguez, Antonio Rodríguez-Bertos

**Affiliations:** 1 VISAVET Health Surveillance Centre, Complutense University of Madrid, 28040 Madrid, Spain; agusrebo@ucm.es (A.R.-M.); cbarcena@ucm.es (C.B.); fjmayoral@ucm.es (F.J.M.-A.); juan.lomillos@uchceu.es (J.M.L.); lucasdo@visavet.ucm.es (L.D.); arbertos@visavet.ucm.es (A.R.-B.); 2 Department of Internal Medicine and Animal Surgery, Faculty of Veterinary Medicine, Complutense University of Madrid, 28040 Madrid, Spain; 3 Department of Animal Production and Health, Veterinary Public Health and Food Science and Technology, Veterinary Faculty, Universidad Cardenal Herrera-CEU, CEU Universities, 46113 Valencia, Spain; 4 Department of Animal Health, Faculty of Veterinary Medicine, Complutense University of Madrid, 28040 Madrid, Spain

**Keywords:** intestinal health, biscuit flour, olive oil by-product, fermented defatted “alperujo”-FDA, histology

## Abstract

**Simple Summary:**

Spanish production of compound feed is among the most important in the Member States of the European Union for all livestock species. However, due to the environmental impact of this large-scale production system, it is important to focus on sustainability, promoting initiatives such as the use of by-products from the food industry applied to animal feed. In this study, laying hens received two types of dietary supplement: biscuit meal, which is a co-product of the human food industry commonly used in the manufacture of compound feed, obtained from the recycling of wasted or expired food products; and fermented defatted “alperujo”, a by-product of modified olive oil, which contain numerous substances with beneficial properties for intestinal health. Hens co-administered with these supplements showed increased intestinal villi development, resulting in improved health. In conclusion, these by-products can contribute to the prevention of intestinal diseases, as well as to the reduction of environmental pollution.

**Abstract:**

In this study, the effects of co-administration with biscuit flour and fermented defatted “alperujo” (FDA) on gut health were evaluated in a batch of laying hens (Hy-Line 2015) on a commercial farm. Animals were divided into two groups: control group and treatment group; and histological and morphometric analyses of all sections of the intestine (duodenum, jejunum, ileum, cecum and rectum) were performed at 10, 18, 25, 50 and 75 weeks of age. During the whole productive period, a decrease in the mortality rate (*p* = 0.01) was observed in treated hens, as well as an increase in the number of eggs produced (*p* < 0.001), their size (*p* < 0.025), and weight (*p* < 0.024). In the early and late stages of production (10, 18 and 50 weeks), a significant increase (*p* < 0.001) in the height and depth of the intestinal villi was observed in the treatment group. Villi height was also significantly higher (*p* < 0.001) in the treatment group up to week 50 in the cecum, and at weeks 18 and 50 in the rectum. We concluded that an economical and sustainable feeding system with less environmental impact, such as co-supplementation with biscuit flour and FDA, could maintain gut health without negatively impacting laying hens’ productive performance.

## 1. Introduction

Advances in animal feed have been significant and have gone hand in hand with the growth in livestock production, especially as far as the intensive livestock industry is concerned. This has led to an increase in the demand for animal feed and changes in the animal feed industry [1,2,3]. The Spanish production of compound feed is among the highest of European Member States for all livestock species. The approximately 37 million tons produced from 2018 to 2019 placed Spain as the leader in animal feed production in the European Union for the first time [1,3]. However, there is a large environmental impact generated by this large-scale production system. Therefore, it is important to raise awareness and promote the use of by-products from the food industry applied to animal feed to ensure sustainability and move towards a circular economy [1,2,3,4].

In recent years, the use of flours in the production of animal feed has been fundamental. Various types of commercial flour exist, with corn flour being predominant in poultry diets. Wheat can be a more economical alternative, although its energy content is slightly lower. In addition, wheat provides more protein, as it contains a variety of essential amino acids such as lysine, methionine, arginine, phenylalanine, and tryptophan [5,6,7,8]. Previous studies have reported the benefits of the controlled addition of various flour types and proportions in animal feed for laying hens [5,9,10,11]. Combined with certain enzymes, such as xylanase, these flours have proved to be useful for improving diet efficiency and egg quality parameters [5,11]. Moreover, certain types of flour can promote greater productive efficiency and stimulate the antioxidant defence mechanism system, improving the immune response and, therefore, inducing beneficial properties for animal health in laying hens [12,13].

Biscuit flour is a co-product of the human food industry commonly used in manufacturing compound feed [4,14]. It is obtained from recycling wasted or expired food products, which are well conserved and preserved, and free from chemical or microbiological contamination [4]. Furthermore, the use of co-products is considered a more economical alternative to using noble-raw materials, such as cereals and fats, since the technological process used was designed to avoid contamination by plastics and packaging in the final product [4]. However, while the main ingredient of this compound is wheat flour, its composition varies widely depending on the supplier, the season and the original raw materials [4,14,15]. Four types of products are used by manufacturers to produce biscuit flour: starchy materials, fatty and sugary components, very fatty fried materials with over 30% ethereal extract, and extra-sweet substances [4]. Biscuit flour is thus a product rich in fat (7–10%) and carbohydrates (55–62% of starch, plus sugars) and low in protein (8–11%), although the availability of amino acids is high [4,14]. In this way, an inexpensive, high-energy and palatable ingredient is achieved [4].

The combination of this co-product with other by-products derived from the human food industry could complement the nutritional deficiencies of a single product and potentially reduce waste and environmental contamination. More specifically, the use of olive oil by-products in animal feed has demonstrated antioxidant, anti-inflammatory and antimicrobial proprieties [16]. Additionally, it contains a significant proportion of non-soluble fibres, polyphenols, oleic acid, carbohydrates, fats and high-quality proteins and polyphenols, which are beneficial for intestinal function [17]. In this study, “alperujo” was first subjected to a fermentation process to stabilize the raw material, hydrolysis to reduce the total fat content, and a drying and milling process to adapt it to animal feed, obtaining fermented defatted “alperujo” (FDA). This by-product has proven to increase intestinal villi height and crypt depth, which translates to an improvement in the absorption of nutrients, thus enhancing gut health in laying hens [18].

We therefore assessed the inclusion of two by-products of the human food industry in laying hens: biscuit flour and FDA. We focused on testing the enhanced capacity of the intestine for absorption in supplemented animals and possible changes in the intestinal mucosa morphology. Healthy gut development could favour the control of intestinal pathogens within the microbiota, thus reducing the incidence of production diseases.

## 2. Materials and Methods

### 2.1. Ethical Approval

This project was carried out in accordance with animal welfare standards for the species. Experimental procedures were approved by the University Complutense of Madrid Animal Care and Ethics Committee (PROEX 152/19).

### 2.2. Animals, Rearing Conditions and Diet

In a commercial farm with an intensive housing system, a whole batch of Hy-Line 2015 laying hens (*n* = 128,535) were randomly divided into two groups: control (*n* = 63,400) and supplemented (*n* = 65,135) and raised from hatching to the end of the production process (75 weeks). The animals were housed according to the Directive 98/58/EC on the protection of animals kept for farming purposes, and the Royal Decree 3/2002, of 11 January 2002, establishing the minimum standards for the protection of laying hens. Briefly: laying hens were housed in cages of a minimum surface of 2000 square centimetres and 20 centimetres high. The minimum space per hen was 750 square centimetres. The cages consisted of a nest, litter, perches, feeders, and drinkers. The two groups were physically separated but shared the same environmental conditions of 24–32 °C, depending on the phase, and 50–70% humidity in the same house. Hens had ad libitum access to feed and water and were monitored once a day during the experiment.

Both control and supplemented animals were fed with a commercial formulation based on barley, corn and soybean. In the supplemented group, biscuit flour was progressively added to the feed until the end of the experiment, as described: 4% in the fifth week of life, 6% in the seventh, and 5% from the ninth week until the end of production. Biscuit flour composition was determined by Labocor S.L. (Colmenar Viejo, Spain) (Table 1). FDA, whose composition has been previously described by Rebollada-Merino et al. [18] (Table 2), was included at 2%. The determination of proteins (MicroKjeldahl method), fat (Soxhlet method) and ash were carried out by the method of the Official Methods of Analysis (OMA). For the determination of crude fibre, the in vitro digestion method (acid–base) was used for crude fibre determination. Moisture was determined by the Karl Fischer method and soluble carbohydrates were calculated by difference (centesimal composition).

Table 3 shows the raw materials of which the treatment diet was composed during the different phases, as the proportion of biscuit flour changed. These ingredients were adjusted in order to achieve an equivalent proportion of nutrients and energy level in both the control and treated groups (Table 4). The control diet contained the same ingredients, adjusting the lack of biscuit flour and FDA by an equivalent increase in the proportion of cereals with the end to reach the same total metabolic energy.

### 2.3. Productive Performance

Production parameters were recorded using Microsoft Dynamics NAV ERP software. The percentage of mortality, the number of eggs produced and the consumption of feed per animal, among other productive parameters (mortality (%), laying (%), feed/hens (g), egg weight (g), egg mass (g/d), egg size (%), dirty eggs (%), broken eggs (%) and commercial total eggs (number)), were recorded from week 19 to week 50 of life (32 weeks in total). The conversion index (CI) per batch was calculated as the amount of feed consumed by the batch during the production period and divided by the total eggs produced by the batch.

### 2.4. Postmortem Study and Sample Collection

On weeks 10, 18, 25, 50 and 75, 15 animals randomly selected from the control group and 15 from the supplemented group (*n* = 150 in total) were sedated intramuscularly with diazepam and euthanized intravenously with an overdose of sodium pentobarbital. A complete postmortem survey was performed on 150 animals. Samples of the duodenum, jejunum, ileum, cecum and rectum were collected and fixed in a 10% formaldehyde commercial buffered solution (Panreac Química SLU, Barcelona, Spain).

### 2.5. Histomorphometric Study

Intestinal samples were processed for histopathology and stained with haematoxylin-eosin, as described elsewhere [18]. Multiple sections of each intestinal segment (duodenum, jejunum, ileum, cecum, and rectum) were assessed under a light microscopy and a histomorphometric evaluation was performed using an image analyzer (Leica Application Suite v.4, Leica Wetzlar, Germany). For each animal, a minimum of 80 intact and well-oriented villi in each intestinal segment: duodenum, jejunum, ileum, cecum and rectum, were measured, as well as a minimum of 80 crypts in each intestinal segment (duodenum, jejunum and ileum).

### 2.6. Statistical Analysis

A statistical study of the productive data and histopathology measurements was performed employing a Mann–Whitney test using IBM SPSS statistics software v25 (IBM, Armonk, NY, USA). Statistical significance was considered at *p* < 0.05.

## 3. Results

### 3.1. Productive Performance

Laying (%) in supplemented animals was 4% superior to the control group; however, the statistical study did not reveal significant differences. Total eggs produced in the supplemented group showed significant differences (*p* < 0.05), with approximately 1,000,000 more eggs produced compared to the control group, as well as an increase in size (*p* < 0.025) and weight (*p* < 0.024). However, broken and dirty eggs were observed to increase significantly in the supplemented group (*p* < 0.001). The rest of the productive parameters did not reveal significant differences between the two experimental groups. Productive performance results are detailed in Table 5.

### 3.2. Histomorphometric Study

**Figure 1:**
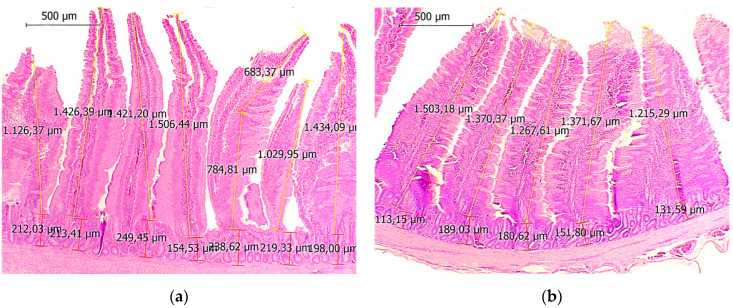

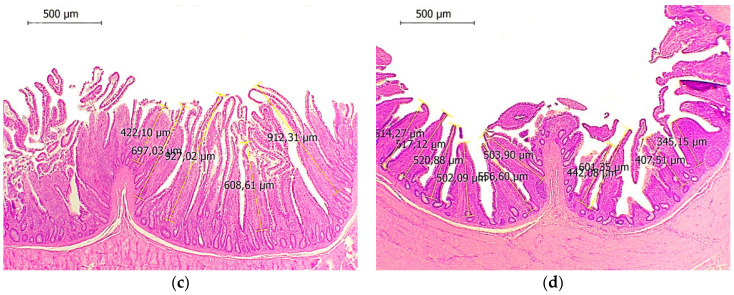
Histomorphometric images of 50-week-old laying hens of a supplemented (left) and a control group (right). The duodenal villi height and the crypt depth were significantly deeper in the supplemented group (**a**) compared to controls (**b**). In the rectum, villi were significantly higher in the supplemented group (**c**) compared to controls (**d**). Hematoxylin-eosin stain, 40×.

A histomorphometric study in the duodenum revealed a significant increase in villi height and crypt depth, mainly in the early stages (Figure 1). In the jejunum, villi height and crypt depth were higher for hens supplemented at intermediate and late stages. Villi of the ileum were significantly higher in early and late stage supplemented animals, while crypts were significantly deeper only in the late stages. The values of the villi of the cecum and rectum of the supplemented group were significantly higher in early and late stages (Figure 1). Comparative histomorphometric results are detailed in Table 6.

## 4. Discussion

Diet is the most important factor in poultry production, and flours are an economical and nutritional supplement that provide high amounts of carbohydrates and protein [1,2,3,4]. Administration of biscuit flour has proven to be an economic solution in the formulation of feed for broilers, partially replacing high-cost cereals with a “non-traditional” raw material that arises from recycling products for human consumption. Biscuit flour can also provide a large amount of energy, due to its high level of carbohydrates and fats, without negatively affecting production parameters [14]. Despite this, to our knowledge, its use has not been previously evaluated in laying hens.

In layers, the beneficial health effects of different flours have been studied, such as açai flour (*Euterpe oleaceae*) or grape pomace flour. Those flours contain phenolic compounds and anthocyanins that have antioxidant effects, as well as a high content of lipids, proteins and fibre [12,13]. In order to compensate for the lack of fibre and antioxidant compounds in biscuit flour, FDA was also administered. This modified olive oil by-product contains phenols and polyphenols with antioxidant activity and fibre. It has also been shown to improve intestinal health, leading to an improvement in productivity [18]. Our study has focused on the evaluation of intestinal health through histomorphometry in order to offer a broader view, considering all sections of the intestine. This method was conducted during all phases of growth, which has not been described to date in any study evaluating hen diets. We suggest this methodology be applied in the evaluation of intestinal health in poultry, as it can provide valuable information regarding all intestinal segments that carry out different functions in digestion and immunity.

The major intestinal villi functions are digestion and absorption. An increase in the height of the villi has been linked to an increase in the absorption surface, although this also depends on the expression of surface enzymes and nutrient transport systems [19,20]. In addition, an increase in the depth of crypts has been related to a major nonspecific immune response against possible superficial lesions in the intestinal epithelium and better efficiency in epithelial renewal [21]. Zhu et al. showed that the administration of rapeseed press-cake, a by-product of rapeseed oil production, in the diet of 33-week-old laying hens caused an increase in the depth of the crypts at the duodenum [22]. In our study, it was observed that the administration of biscuit flour and FDA significantly increased, in first place, the height of the villi of the duodenum at 10, 25 and 50 weeks, the jejunum at 25 weeks, and the ileum at 10, 50 and 75 weeks. Secondly, we observed a significant increase in the crypt depth of the duodenum at 10 and 18 weeks, the jejunum at 50 weeks, and the ileum at 50 and 75 weeks.

These results are similar to those of a previous study that found that FDA increased crypt depth and duodenal villus height in chickens up to 16 weeks old [18]; however, in those treated with biscuit flour and FDA, there is an improvement in the results compared to those treated only with FDA, especially during the hatchery phase, with more than 3% higher growth. These effects on the duodenum could be related to the antioxidant effect of phenols and polyphenols and the high fibre content of “alperujo”.

In poultry, the cecum plays a central role in fermenting and digesting food, producing a wide variety of fatty acids and vitamin B, and contributing to the reabsorption of water. Nevertheless, much of the research on the effect of dietary supplements on the gut health of birds does not include a histomorphometric analysis of the cecum or does not find significant differences [23]. The results presented here indicate an increase in cecum villi height at all evaluation stages, except for week 18. These results are similar to those previously indicated in layers after supplementation with FDA: increased height up to 16 weeks and after 24 weeks of age [18]; but, when comparing it directly with the results of the treatments with FDA only, a considerable improvement is observed, especially in the hatchery phase (10 weeks) and phase II (50 weeks).

A recent study highlights the importance of diet and growth stage in the morphometric development of the rectum in broilers, observing a progressive increase in the growth of colorectal villi, both in width and height [24]. Despite the fact that the productive life of broilers and chickens varies significantly, we observed a greater height of late-stage colorectal villi in layers (50 and 75 weeks old), similar to what was reported in the final stages of broiler production (44 days).

The small intestine plays a key role in calcium absorption [25,26,27,28]. In birds, the greatest activity of the two types of calcium transport mechanisms, transcellular and paracellular, occurs primarily in the jejunum and duodenum [29]. In our investigation, we determined that broken eggs significantly increased in the treatment group; unlike in previous studies [18], in which supplementation with FDA in layers showed a significant decrease in the percentage of broken eggs. This suggests the need to revise calcium content after biscuit flour supplementation and to further investigate the influence of biscuit flour on calcium metabolism in laying hens. Cereal flours in combination with certain enzymes, such as xylanase, increased the thickness of the shell and the mass of the egg, although effects at the productive level were not reported [5,8,11].

We reported a significant increase in laying percentage, which is 3% higher than that obtained in the study by Rebollada-Merino et al. On the other hand, an increase in the number of eggs produced is also achieved, as well as an increase in weight and size. This could be related to an improvement of intestinal health in the first weeks of life, which may favour the absorption of nutrients that are provided in the diet throughout the production phase. In fact, as previously suggested, an increase in the height of the villi during the first weeks of life influences the digestive function during later production phases [18,30]. On the other hand, contrary to the study by Rebollada-Merino et al., the treatment group presented significantly lower mortality, which could be related to the antioxidant and systemic anti-inflammatory effects of phenols and polyphenols present in FDA [18].

Dirty eggs are related to factors such as the age of the hen, pathology and stress, facilities, management systems, hygiene, and nutrition [31]. We observed an increased number of dirty eggs in the treatment group, which may be due to biscuit flour’s high content of monosaccharides and FDA’s high percentage of fibre. However, even if FDA contains soluble fibre, FDA alone did not exert any negative effects regarding dirty eggs in previous studies [18]; in fact, it reduced the percentage of dirty eggs in the group supplemented with FDA. High wheat content in the biscuit flour may have also contributed, as an increase in the number of dirty eggs in wheat-based versus corn-based diets has been reported [8].

## 5. Conclusions

The combined administration of biscuit flour and FDA seemed to maintain a significant increase in the height of villi and the depth of crypts, especially in the early stages. This may improve digestive function, resulting in an increase in egg production, with greater weight and size. Furthermore, the increase in the depth of intestinal crypts may contribute to intestinal epithelial renewal and the development of a nonspecific immune response to harmful situations. This can influence intestinal and systemic health, with a significant reduction in the mortality index of treated hens observed. To summarise, co-administration with biscuit flour does not negatively impact health and productive performance in laying hens, and could also be a more economical and sustainable feeding system with a lower environmental impact.

## Figures and Tables

**Table 1:** Biscuit flour nutrient composition.

Calculated Nutrient Content	Diet
Moisture 103° (%w.w.) ^1^	9.7
Ash content (%w.w.)	2.2
Crude fat (%w.w.)	10.9
Crude protein (%w.w.)	10.3
Carbohydrates (%w.w.)	44.7
Sugars (%w.w.)	12.1
Crude fibre (%w.w.)	1.0

^1^ %w.w. = % wet weight.

**Table 2:** Fermented defatted “alperujo” nutrient composition.

Calculated Nutrient Content	Diet
Moisture 103° (%w.w.)	12.2
Crude protein (%w.w.)	6.4
Crude fat (%w.w.)	3.0
Ash content (%w.w.)	7.7
Lignin (%w.w.)	23.3
Acid detergent fibre (%w.w.)	39.2
Neutral detergent fibre (%w.w.)	49.3
Tannins (%w.w.)	0.06
Oleic acidity index (%w.w.)	46.1
Peroxide value (%w.w.)	7.9
Total polyphenols (meq/kg)	0.89
Crude fibre (%w.w.)	27.7

**Table 3:** Ingredient composition of the control and the supplemented groups: 4%, 5% or 6% biscuit flour (BF) inclusion.

Ingredient (g/kg)	Diet (Control) ^1^	Diet (4% BF) ^2^	Diet (5% BF) ^3^	Diet (6% BF) ^4^
Yellow corn (grain)	442.00	170.31	432.40	380.66
Barley E 11, 4	225.00	440.70	165.70	200.00
Soybean meal 47	200.00	170.80	177.44	185.19
Sunflower meal 28	120.00	120.00	120.00	120.00
Biscuit flour	-	40.00	50.00	60.00
FDA (fermented defatted “alperujo”)	-	19.48	19.48	19.48
Calcium carbonate	14.10	12.83	12.83	12.83
Tricalcium phosphate	7.90	8.23	8.23	8.23
Soybean oil	9.00	9.00	5.00	5.00
Premix	4.00	4.00	4.00	4.00
Common salt, NaCl	1.97	1.92	2.19	1.89
Sodium bicarbonate	1.98	1.10	1.10	1.10
L-lysine 95%	1.03	0.96	0.96	0.96
Methionine	0.75	0.67	0.67	0.67

^1^ Control group diet. ^2^ Supplemented group diet with 4% biscuit flour and 2% FDA in the fifth week of life. ^3^ Supplemented group diet with 5% biscuit flour and 2% FDA in the seventh week of life. ^4^ Supplemented group diet with 6% biscuit flour and 2% FDA from the ninth week until the end of production.

**Table 4:** Calculated nutrient composition of the diet.

Calculated Nutrient Content	Diet
EMT Energy (kcal/kg)	2700
Protein (%w.w.)	17
Fat (%w.w.)	3
Linoleic acid (%w.w.)	1
Fiber (%w.w.)	5
Calcium (%w.w.)	0.9
Digestible phophorus (%w.w.)	0.34
Sodium (%w.w.)	0.5
Chlorine (%w.w.)	0.16
Potassium (%w.w.)	0.5
Digestible methionine (%w.w.)	0.34
Digestible lysine (%w.w.)	0.8
Tryptophan (%w.w.)	0.21

**Table 5:** Production performance of control and supplemented group.

Productive Parameter	Control	Supplemented	*p*-Value ^1^
Mortality (%)	0.22	0.13	0.010
Laying (at peak) (%)	80.73	84.91	0.083
Feed/hens (g)	110.63	116.17	0.227
Egg weight (g)	60.23	60.33	0.024
Egg mass (g/d)	48.97	51.39	0.025
Extra-large eggs (%)	2.03	2.35	0.320
Large eggs (%)	33.81	33.95	0.564
Medium eggs (%)	51.15	49.68	0.398
Small eggs (%)	10.11	10.53	0.990
Dirty eggs (%)	0.80	0.84	<0.001
Broken eggs (%)	0.89	1.16	0.007
Total eggs (number)	11,125,503	12,115,667	<0.001
Total eggs (Kg)	674,109	734,441	<0.001
CI (Conversion Index)	0.02	0.02	1.000

^1^ A Mann–Whitney test was used to assess significant differences between control (*n* = 64,300) and supplemented (*n* = 65,135) animals (*p* < 0.05).

**Table 6:** Histomorphometric results in the intestinal segments. Number of samples (N), mean values (Mean), standard deviation (SD) and *p*-value are detailed per age group.

	Control			Supplemented			*p*-Value ^1^
	N	Mean	SD	N	Mean	SD	
**Duodenum villi height**							
10 weeks	15	1231.03	305.57	15	1433.68	281.04	<0.001
18 weeks	15	1346.91	272.01	15	1263.38	324.73	0.058
25 weeks	15	1385.55	300.30	15	1475.21	327.92	0.047
50 weeks	15	1251.71	356.35	15	1381.29	413.24	0.011
75 weeks	15	1385.50	281.82	15	1324.54	340.35	0.156
**Duodenum crypt depth**							
10 weeks	15	220.66	86.70	15	268.69	70.90	<0.001
18 weeks	15	202.82	54.23	15	279.72	87.52	<0.001
25 weeks	15	260.66	79.49	15	252.50	74.55	0.290
50 weeks	15	419.44	126.08	15	406.68	114.12	0.362
75 weeks	15	348.77	119.90	15	337.39	203.08	0.011
**Jejunum villi height**							
10 weeks	15	1034.71	327.79	15	852.90	227.81	<0.001
18 weeks	15	991.87	189.33	15	893.39	189.54	<0.001
25 weeks	15	1021.71	273.43	15	1145.86	319.48	0.001
50 weeks	15	980.07	247.46	15	1034.85	226.63	0.052
75 weeks	15	984.48	278.55	15	1043.11	245.76	0.072
**Jejunum crypt depth**							
10 weeks	15	168.77	57.68	15	144.41	40.75	0.005
18 weeks	15	167.57	44.07	15	177.14	49.60	0.089
25 weeks	15	198.75	76.46	15	206.96	63.53	0.070
50 weeks	15	230.35	78.76	15	263.35	76.30	<0.001
75 weeks	15	201.12	59.08	15	209.38	89.98	0.883
**Ileum villi height**							
10 weeks	15	482.98	118.89	15	664.41	206.66	<0.001
18 weeks	15	680.30	194.25	15	604.60	170.64	0.001
25 weeks	15	708.90	164.20	15	649.25	167.02	0.005
50 weeks	15	669.85	198.77	15	873.49	189.47	<0.001
75 weeks	15	762.64	184.29	15	850.90	219.08	0.001
**Ileum crypt depth**							
10 weeks	15	121.28	35.54	15	190.00	169.81	0.256
18 weeks	15	130.55	35.50	15	126.58	40.03	0.418
25 weeks	15	131.03	32.53	15	143.88	50.79	0.118
50 weeks	15	150.06	40.78	15	210.51	61.39	<0.001
75 weeks	15	167.47	50.54	15	141.12	37.09	<0.001
**Cecum vill height**							
10 weeks	15	264.35	121.46	15	409.08	166.48	<0.001
18 weeks	15	320.37	280.83	15	307.95	177.40	<0.001
25 weeks	15	115.42	40.13	15	132.67	43.29	<0.001
50 weeks	15	256.21	166.96	15	642.49	408.55	<0.001
75 weeks	15	408.33	318.43	15	549.75	425.84	0.101
**Rectum villi height**							
10 weeks	15	416.15	147.77	15	437.73	141.26	0.313
18 weeks	15	459.59	150.96	15	542.52	192.01	<0.001
25 weeks	15	166.19	59.69	15	152.22	46.99	0.100
50 weeks	15	476.45	166.06	15	562.33	190.41	<0.001
75 weeks	15	522.29	136.79	15	579.13	189.48	0.011

^1^ A Mann–Whitney test was used to assess significant differences between control and supplemented animals (*p* < 0.05).

## Data Availability

Data are contained within the article.
